# Recent advances in engineering nanotopographic substrates for cell studies

**DOI:** 10.3389/fbioe.2022.1002967

**Published:** 2022-09-06

**Authors:** Ignasi Casanellas, Josep Samitier, Anna Lagunas

**Affiliations:** ^1^ Nanobioengineering Group, Institute for Bioengineering of Catalonia (IBEC), Barcelona Institute of Science and Technology (BIST), Barcelona, Spain; ^2^ Department of Electronics and Biomedical Engineering, Faculty of Physics, University of Barcelona (UB), Barcelona, Spain; ^3^ Biomedical Research Networking Center in Bioengineering, Biomaterials, and Nanomedicine (CIBER-BBN), Madrid, Spain

**Keywords:** receptor nanoclustering, nanofabrication, nanotopography, nanopatterning, cell response

## Abstract

Cells sense their environment through the cell membrane receptors. Interaction with extracellular ligands induces receptor clustering at the nanoscale, assembly of the signaling complexes in the cytosol and activation of downstream signaling pathways, regulating cell response. Nanoclusters of receptors can be further organized hierarchically in the cell membrane at the meso- and micro-levels to exert different biological functions. To study and guide cell response, cell culture substrates have been engineered with features that can interact with the cells at different scales, eliciting controlled cell responses. In particular, nanoscale features of 1–100 nm in size allow direct interaction between the material and single cell receptors and their nanoclusters. Since the first “contact guidance” experiments on parallel microstructures, many other studies followed with increasing feature resolution and biological complexity. Here we present an overview of the advances in the field summarizing the biological scenario, substrate fabrication techniques and applications, highlighting the most recent developments.

## 1 Introduction

**FIGURE 1:**
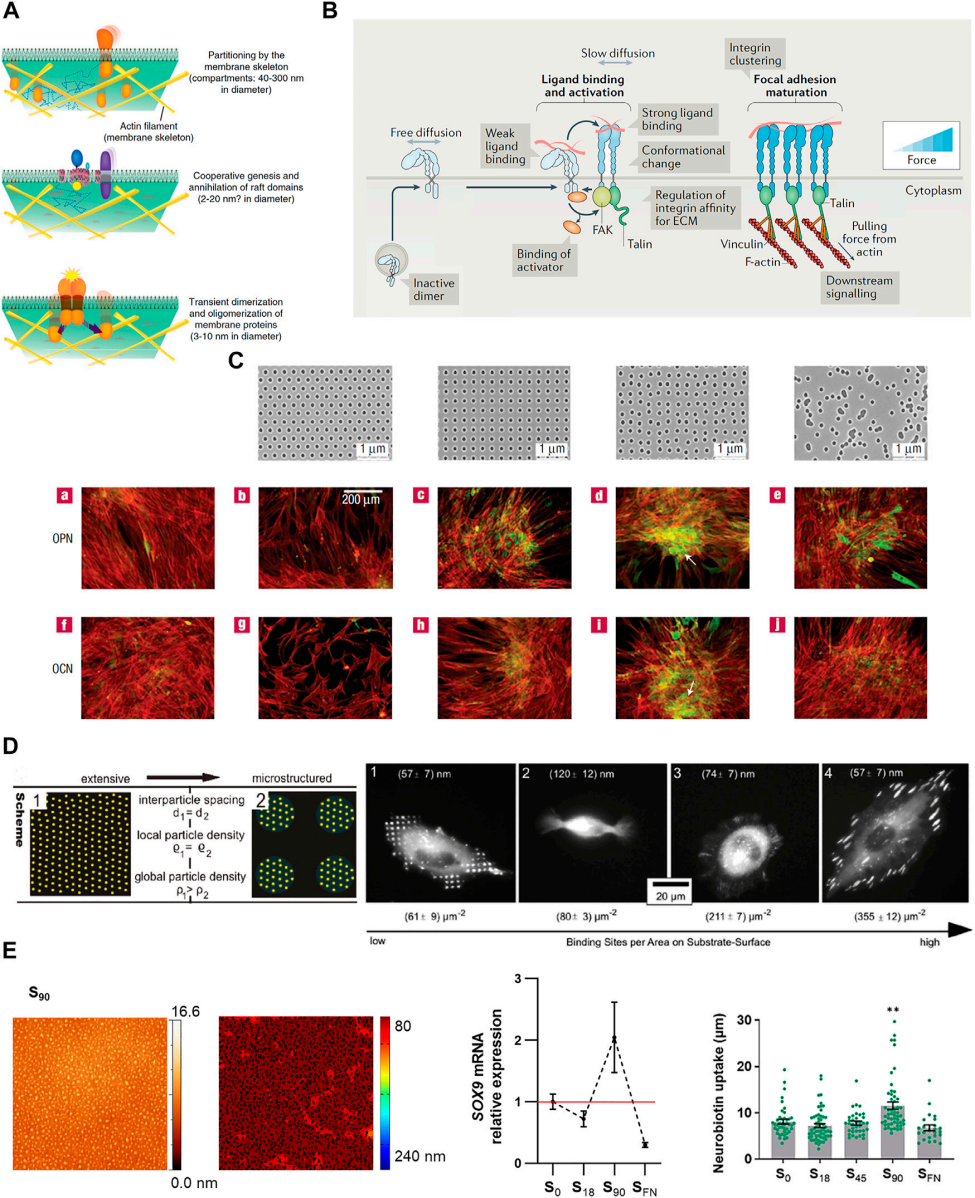
**(A)** Hierarchical organization of cell membrane compartments. Reprinted with permission from Kusumi et al., 2011. Copyright 2011 Elsevier. **(B)** Receptor nanoclustering: Ligand-mediated integrin clustering initiates the recruitment of adaptor proteins at FAs, leading to cytoskeleton engagement, force transmission and downstream signaling activation. Adapted with permission from Kechagia et al., 2019. Copyright 2019 Springer Nature. **(C)** Osteoprogenitor differentiation (osteopontin (OCP) and osteocalcin (OCN) expression, and bone nodule formation (white arrows)) on nanotopographies with different levels of disorder, fabricated by EBL. Reprinted with permission from Dalby et al., 2007b. Copyright 2007 Springer Nature. **(D)** BCML combined with photolithography were used to create micro- and nanopatterned surfaces of the cell adhesive ligand cyclic-(RGDfK). The development of stable FAs, number, size and adhesion strength is more influenced by local than global ligand density. Adapted with permission from Deeg et al., 2011. Copyright 2011 American Chemical Society. **(E)** The nanopatterning of RGD functionalized dendrimers revealed a threshold nanopattern configuration to induce cell response promoting chondrogenic differentiation and enhancing GJIC. Adapted from Casanellas et al., 2020 and Reprinted with permission Casanellas et al., 2022. Copyright 2022 Future Medicine Ltd.

The compartmentalization of cellular functions is a ubiquitous strategy to increase efficiency, providing spatio-temporally discrete domains for dynamic processes to take place simultaneously, in close vicinity, and without interfering with each other. The plasma membrane is generally accepted as being compartmentalized (Garcia-Parajo et al., 2014; Nicolson, 2014). This characteristic emerges from the temporary limitation of lateral diffusion, promoting confinement and allowing lipids and proteins to be organized in specific locations of variable size and composition (Kusumi et al., 1993; Jacobson et al., 2019). Restrictions in lateral diffusion of membrane components have been mainly attributed to their association to the underlying cytoskeleton as described by the “membrane skeleton fence model”, in which fences or corrals are defined by transmembrane proteins acting as posts linked to the cytoskeletal structures (Kusumi and Sako, 1996; Kusumi et al., 2012). Also, the presence of extracellular lattices (Lajoie et al., 2009), or specific lipid domains or “lipid rafts” (van Meer et al., 1987) can create regions of restricted lateral diffusion in the plasma membrane. This compartmentalization is hierarchically organized from small nanoclusters of dynamic small protein oligomers (of 3–10 nm diameter) and lipid rafts (2–20 nm) to actin-cytoskeletal fence domains (40–300 nm) up to bigger domains of several micrometers, thereby allowing the multiscale regulation of the membrane protein function (Kusumi et al., 2011) (Figure 1A).

The cellular microenvironment is also organized at the nanoscale, as seen for collagen, the main structural protein in the extracellular matrix (ECM). Collagen I is assembled by three peptide chains of collagen that conform a helical structure of around 1.5 nm diameter and 300 nm length, which then organize into microfibrils with a cross-section of around 3 × 5 nm (Jiang et al., 2004). The ECM protein fibronectin forms bundles of fibrils, in which the average span of a fibronectin molecule (a dimer of two polynucleotides) in each fibronectin fibril is ∼92 nm (Früh et al., 2015). This favors ligand interaction with the receptors at the cell membrane in a particular configuration that is confined to the nanometer scale.

Therefore, nanotopography represents an effective physical approach for studies on cell behavior mediated by cell-cell environment interactions. Nanotopographies (1–100 nm) lie in the same scale range as many ECM proteins, allowing the direct interaction of the material with single cell receptors and their nanoclusters. Nanoscale surface topography affects cellular and tissue responses, including adhesion, migration, growth, morphogenesis, and differentiation (Martínez et al., 2009; Luo et al., 2022).

## 2 Receptor nanoclustering

Specific protein-protein and protein-lipid interactions promote oligomerization, aiding the formation of signaling complexes at the cell membrane. Glycosylphosphatidyl-anchored proteins (GPI-APs) are a class of soluble proteins attached to the external side of the plasma membrane. They form small clusters of up to four molecules (<5 nm) stabilized in sphingolipid- and cholesterol-dependent domains or rafts. These lipid rafts act as sorting platforms for the GPI-APs selective delivery to the apical membrane in polarized epithelial cells, where they exert specialized functions (Zurzolo and Simons, 2016). Besides lipid-linked proteins, many transmembrane proteins also cluster to exert their functions.

The clustering of transmembrane receptors is common among different types of immune cells (Dustin and Groves, 2012; Li and Yu, 2021). T cell receptors (TCRs) on resting T cells can be found as monomers and as cholesterol- and sphingomyelin-stabilized nanoclusters (<10%) containing 2–30 TCRs each (Molnár et al., 2012). Upon activation of TCRs, they assemble into larger clusters of ten up to hundreds of receptors, which recruit kinases and adaptor proteins including Lck, ZAP-70, Lat, and SLP76. These microclusters initiate and sustain TCR signaling at the immunological synapse. Moreover, TCR microclusters associate and are transported by cortical F-actin flows over micrometer distances along the synapse (Dustin and Groves, 2012; Yi et al., 2019; Balagopalan et al., 2021). The enrichment of oligomeric TCRs has been reported to increase the sensitivity of memory T cells compared to naïve T cells (Kumar et al., 2011). A similar hierarchical organization has been described for integrin receptors. Integrins are transmembrane receptors that mediate cell-cell and cell-extracellular matrix (ECM) adhesion (Kechagia et al., 2019). However, integrin binding alone is insufficient to elicit full adhesion. Instead, upon ligand binding, integrin receptors arrange into nanoclusters that build tension through the recruitment of adaptor proteins such as paxillin, vinculin, talin, FAK or SRC, cytoskeletal engagement into focal adhesions (FAs) and downstream signaling activation (Hu et al., 2015; Kechagia et al., 2019) (Figure 1B). Remarkably, within FAs, active and inactive β1 integrins segregate into different nanoclusters, thus suggesting integrin activity is not only regulated at the monomeric level but is subjected to collective or coordinate regulation at the level of the nanoclusters (Spiess et al., 2018). As integrins, Eph tyrosine kinase receptors cluster upon the interaction with their ligand, ephrin, which is presented on the surface of neighboring cell membranes. During development, Eph receptors act as positional cues in tissue patterning by regulating cell adhesion and repulsion. Ephrin ligands presented as concentration gradients guide axonal patterning in retinotectal mapping and stem cell migration in the developing intestines (Klein, 2012). Activation of Eph receptors occurs immediately after ligand interaction, inducing receptor polymerization. Maximum receptor activation is reached on clusters of five to eight receptors, after which oligomers cannot grow further by recruiting more monomers and instead, they grow through the condensation of oligomers into larger complexes that dampen the signaling. These polymerization–condensation dynamics provide a framework for the mechanism by which cells properly respond to variable concentrations and gradients of the ephrin ligand (Ojosnegros et al., 2017).

## 3 Nanofabrication

From the first contact guidance experiments (Curtis and Varde, 1964), microfabrication techniques first developed for the electronics industry came into use to produce micro- and nanopatterned surfaces for cell studies, with high feature resolution and increased biological complexity. Fabrication methods to produce controlled nanotopographies on cell culture substrates mainly rely on the use of lithographic techniques, which in general require specialized equipment and skilled personnel, thus limiting their widespread application. The later introduction of the directed self-assembly techniques to produce nanostructured surfaces greatly facilitated the implementation of nanotopography production in general wet labs. Here we summarize the main fabrication techniques used to generate nanotopographical substrates, and their characteristics.

### 3.1 Photolithography

Photolithography or optical lithography is a patterning technique in which a light-sensitive chemical (photoresist) coated on the substrate is selectively exposed to light through a mask. The photoresist either collapses or hardens in the regions exposed to light and the pattern emerges on the substrate by dissolving the softer parts of the coating, which can subsequently be transferred to the substrate material. The wavelength of the light used determines the minimum feature size that can be impressed on the photoresist: The use of incoherent, vacuum ultraviolet (VUV) radiation of 172 nm allowed the production of nanoscale features with a minimum lateral feature size of 350 nm (Mironov et al., 2020). To overcome the resolution limitations, surface plasmon polaritons (SPPs), able to surpass the diffraction-limits, have been used for the fabrication of nanopatterns with a half-pitch resolution of less than 15 nm (Luo and Ishihara, 2004; Dong et al., 2014).

### 3.2 Electron beam lithography and ion beam lithography

In electron beam lithography or e-beam lithography (EBL), a focused beam of electrons is applied (direct-writing) on an electron sensitive coating on a substrate (Lercel et al., 1994). This is a maskless lithography technique in which custom nanopatterns can be transferred to a substrate with up to 3–5 nm resolution (Ermis et al., 2018). Like photolithography, the coating is degraded or crosslinked upon exposure and after a development process, patterns are revealed. While conventional lithography mostly relies on flat wafer-base processing, EBL can be applied on curved surfaces (Lee et al., 2019). However, compared to photolithography, only small areas can be patterned at a time and the equipment manipulation is tough, which makes the whole process significantly slower. Ion beam lithography (IBL) or focused ion beam lithography (FIBL) uses a narrow scanning ion beam source (typically of gallium ions) instead of a focused beam of electrons to pattern a resist. Compared to EBL, IBL offers higher resolution due to ions have much heavier mass than electrons and more momentum, thus leading to smaller wavelengths and reducing diffraction, but also minimizing the back scattering and radiation towards sensitive resists (Joshi-Imre and Bauerdick, 2014; Li et al., 2021).

### 3.3 Scanning probe lithography approaches

SPL approaches are a set of maskless nanolithographic techniques based on the ability of scanning probe microscopy to create variable surface patterns either by adsorption, nanoshaving and/or nanografting (Rosa and Liang, 2009). They include dip-pen nanolithography (DPN), fluidic force microscopy (FluidFM), and polymer pen lithography (PPL), among others. DPN was pioneered by the group of Prof. Mirkin (Piner et al., 1999; Salaita et al., 2007), where an AFM tip is used to create patterns of 15–100 nm by direct writing on the substrate. A molecular ink is transferred from the atomic force microscope (AFM) tip to the substrate by the spontaneous formation of a water meniscus, which is facilitated by the ambient conditions. DPN can work in sequential or parallel modes (multiplexed DPN), where parallel DPN tip arrays are scanned on the substrate simultaneously, thus significantly improving the throughput limitations of the technique (Ma et al., 2018). FluidFM introduces microfluidic channels (300 nm-8 µm) into the AFM probes allowing to dispense volumes of fluid that can be below the femtoliter range. The patterns are created when the nanopipette contacts the surface and the ink is released from the probe with a short pressure pulse (few hPa) (Zambelli et al., 2018). Alternatively, a cantilever-free scanning probe molecular printing technology referred as polymer pen nanolithography (PPL) was introduced to overcome the throughput issues and the use of complicated pen arrays (Huo et al., 2008). Since the SPL techniques work under mild conditions, they allow patterning sensitive compounds such as DNA, proteins, lipids, viruses and even polymers for 3D additive manufacturing (Liu et al., 2022).

### 3.4 Nanoimprint lithography

Nanoimprint lithography (NIL) is a simple and low-cost lithography technique in which a pattern is transferred by mechanical deformation of a polymer resist from a previously nanostructured mold (created by photolithography or EBL). The transfer of the nanopattern can be conducted in several ways: by thermocompression using high temperatures to soften the polymer resist while pressing it with the stamp, also known as hot embossing lithography, or by using UV light to cross-link and harden a soft polymer resist during the imprint (UV-NIL). UV-NIL requires the substrate and/or stamp to be transparent to UV wavelengths (Modaresialam et al., 2021).

### 3.5 Soft lithography

First introduced by Bain and Whitesides in 1989 (Bain and Whitesides, 1989), soft lithography refers to a number of techniques that use elastomeric stamps (typically of polydimethylsiloxane (PDMS)) previously cast on a master, to transfer micro- or nanopatterns to a substrate. It includes replica molding, microcontact printing, micromolding in microcapillaries, microtransfer molding, and solvent-assisted micromolding. Soft lithography is a low-cost technique that does not require stringent control over the environment (such as a clean-room facilities), thus being accessible to general wet labs (Qin et al., 2010).

### 3.6 Directed self-assembly of nanostructures

Supramolecular chemistry can be used as a bottom-up approach to achieve nanopatterned surfaces based on the self-organization of molecular scale architectures, allowing precision on the nanofeature position. Compared to the self-assembly of small molecules, polymers offer higher stability and durability due to their mechanical and physical properties. Self-assembly of block copolymers (BCPs) has attracted considerable attention in nanoscience due to its ability to self-assemble both in bulk and in solution into different types of nanostructures through the repulsion of their immiscible blocks (Mai and Eisenberg, 2012). BCP micelle nanolithography (BCML) has been extensively used to generate ordered and disordered nanopatterns of gold nanoclusters on surfaces with well-controlled interparticle distances (Glass et al., 2003). Dendrimers, presenting a highly branched and easily tunable size and chemical structure, have been used to create nanopatterns with a liquid-like order on low charged surfaces (Lagunas et al., 2014), and DNA and peptides have been used to build nanostructures presenting multiple epitopes with nanoscale spatial control (Stephanopoulos et al., 2015; Wang et al., 2021).

## 4 Biological applications

The first visible phenomena of nanostructures-cell interaction are the changes in cell adhesion, spreading and morphology, which provide cues to predict cellular functions. Studies have explored the influence of different nanotopographies on the adhesive/spreading behavior of various cell types, some of them summarized in Table 1. Nanotopography has been vastly employed to control cell differentiation with especial emphasis on enhancing tissue integration in bone implants (Chen et al., 2018). Due to the easy manufacturing, first attempts were conducted by using the surface roughness strategy. However, the lack of control in the produced structures, and poor reproducibility, prompted the use of lithographic techniques to fabricate nanostructured biocompatible materials that promote osteointegration (Figure 1C and Table 1).

**TABLE 1:** Influence of nanotopographies on cell response.

Technique	Cell type	Cell response	Ref
NIL (Hot embossing)	SMC	Nanopatterned gratings with 350 nm line width, 700 nm pitch, and 350 nm depth in PMMA, produced cell alignment towards the gratings both of nuclei and cell body, elongation, polarization of MTOC towards the axis of cell alignment, and reduced cell proliferation	Yim et al. (2005)
EBL	Human fibroblasts	The nanopits (of 100 nm diameter and 100 nm depth on PMMA) reduced adhesion, spreading and stress fiber formation. Also reduced the nuclear area and there was a closer spacing of centrosomes within the nucleus	Dalby et al. (2007a)
EBL, Hot embossing	Osteoprogenitors from bone marrow samples, hMSCs	120 nm diameter, 100 nm depth, 300 nm mean spacing nanopits in PMMA with different levels of disorder. Highly ordered nanotopographies produce low to negligible cellular adhesion and osteoblastic differentiation	Dalby et al. (2007b)
Soft lithography, Hot embossing	hMSCs	The nanopatterned gratings (350 nm line width, 700 nm pitch and 350 nm in depth in PDMS and TCPS) decreased the expression of integrins and promote an aligned actin cytoskeleton towards the gratings. On the rigid TCPs, gratings (500 nm line width, 1 µm pitch and 350 nm in depth) affect the mechanical properties of the cells	Yim et al. (2010)
DPN	hMSCs	Nanodots with 70 nm diameter, separated by defined spacings of 140–1,000 nm with different terminal groups (carboxyl, amino, methyl and hydroxyl). Spacing and chemistry have different effects on adhesion and stemness maintenance	Curran et al. (2010)
EBL, Hot embossing	MSCs from bone marrow, SaOS2 osteoblasts	Pits of 120 nm diameter, 100 nm depth and a random displacement of ±50 nm, with mean 300 nm pitch in PCL increase cell adhesion in both cell lines and promote osteogenic differentiation through adhesion in MSCs	Allan et al. (2018)
BCML with poly-styrene (PS) homopolymer as an ordering interference reagent	MC3T3-E1 osteoblasts	Integrin clustering depends on the local order of RGD ligands when the global average ligand spacing is larger than 70 nm	Huang et al. (2009)
BCML, photolithography	REF	Cell adhesion more influenced by local (<60 nm ligand spacing) than global ligand density	Deeg et al. (2011)
BCML	HSCs	32 nm maximum ligand spacing for cell adhesion, and lipid raft clustering	Altrock et al. (2012)
BCML	hMSCs	Maintenance of undifferentiated state favored on nanopatterns of 68 nm spacing	Medda et al. (2014)
BCML, transfer lithography	rMSCs	Large (161 nm) nanospacings favor chondrogenic differentiation	Li et al. (2015)
BCML, photolithography, and transfer lithography	hMSCs	Adipogenic and osteogenic differentiation favored on large (95 nm) nanospacings	Wang et al. (2015)
BCML, with poly-styrene (PS) homopolymer as an ordering interference reagent, transfer lithography	Human breast myoepithelial cell line, HUVECs, MEFs, MCF 10A	Integrin clustering and the formation of FAs integrate the effects of ligand spacing and substrate force loading	Oria et al. (2017)
Self-assembled diblock copolymers	HEK293T expressing the EphB2 receptor fused to the fluorescent protein mRuby	Nanopatterns of surface-bound ephrinB1/Fc ligands accelerate receptor oligomerization (receptor monomer polymerization was accelerated by 25–30%)	Hortigüela et al. (2018)
Dendrimer nanopatterning	hASCs	Chondrogenesis and GJIC are enhanced by a nanopattern configuration in which 90% of the surface area presents adhesion sites separated <70 nm, providing an onset for cell signaling	(Lagunas et al., 2017; Casanellas et al., 2022)

SMC: bovine pulmonary artery smooth muscle cells; PMMA: polymethylmethacrylate; MTOC: microtubule organizing centers; hMSCs: human mesenchymal stem cells; PDMS: polydimethylsiloxane; TCPS: tissue culture polystyrene; PCL: polycaprolactone; REF: rat embryonic fibroblasts; HSCs: hematopoietic stem cells; rMSCs: rat mesenchymal stem cells; HUVECs: human umbilical vein endothelial cells; MEFs: mouse embryonic fibroblasts; MCF, 10A: mammary epithelial cells; HEK293T: human epithelial kidney 293 cells; hASCs: human adipose-derived mesenchymal stem cells.

In many cases, the assembly of membrane receptors into fully functional microcomplexes requires of both ligand occupancy and receptor clustering. Spatz and coworkers used BCML to create ordered gold nanopatterns coated with the integrin receptor ligand arginine-glycine-aspartic acid (RGD), present in many ECM proteins. The gold nanodots, of less than 8 nm in diameter, allowed the binding of one integrin per dot and were positioned at different interdot spacings on a non-fouling substrate. Authors observed that a ligand spacing of more than 73 nm impairs integrin clustering, cell adhesion and spreading, and dramatically reduces the formation of FAs and actin stress fibers (Arnold et al., 2004). Since this seminal work, BCML has been used in a number of cell studies, showing the prevalence of local over global ligand density (Deeg et al., 2011) (Figure 1D and Table 1), and that integrin clustering influences many aspects of cell behavior, including cell differentiation (Table 1). More recently, Roca-Cusachs and coworkers used BCML to create cell adhesive nanopatterns on substrates of different rigidity, and they found that the optimal ligand spacing for cell adhesion increases as substrate stiffness decreases (Oria et al., 2017) (Table1).

The multivalent interactions between ligand and receptors, in which the simultaneous binding of multiple ligands on receptor complexes takes place (Kiessling et al., 2006), have been extensively used to study receptor clustering and the downstream signaling in cells. Self-assembled diblock copolymers of polystyrene-blockpoly(methyl methacrylate) (PS-b-PMMA) were used to produce nanopatterned substrates able to establish multivalent interactions between surface-bound ephrinB1 ligands and membrane EphB2 receptors. The preclustering of ephrinB1 ligands in the nanopatterns resulted in a more efficient and faster receptor oligomerization kinetics compared to the traditional cross-linked ligand presentation (Hortigüela et al., 2018). Also, dendrimer nanopatterning of RGD-functionalized dendrimers was used to study the effects of the local RGD ligand density on the adhesion, differentiation, and gap junction intercellular communication (GJIC) of mesenchymal stem cells (Lagunas et al., 2017; Casanellas et al., 2020; Casanellas et al., 2022) (Figure 1E).

## 5 Conclusions and perspectives

Nanoscale cell-environmental interactions regulate cell behavior. Nanotopography produced by lithographic techniques and/or by the self-assembly of molecular scale architectures effectively mimics those interactions, helping to direct particular cell responses and providing information about the underlying mechanisms. We expect that further advances in the field by including stimuli-responsive materials, combined with super-resolution microscopies, will bring more detailed information on the molecular mechanisms that direct cell function, unveiling traits that are normally hidden by the ensemble average in bulk experiments. This will provide an otherwise unavailable insight on the cell interactions at the nanoscale so that they can be used to systematically drive cell responses by fabricating the appropriate nanotopographical substrates, with potential applications in translational medicine.
